# Case Report: rescue thrombolysis after failed primary percutaneous coronary intervention in coronary artery ectasia with ST-elevation myocardial infarction

**DOI:** 10.3389/fcvm.2025.1595445

**Published:** 2025-09-24

**Authors:** Jiejun Sun, Muyun Tang, Zhiyu Zhang, Ming Yang, Zhujun Shen, Ran Tian, Zhenyu Liu

**Affiliations:** ^1^ Department of Cardiology, Peking Union Medical College Hospital, Chinese Academy of Medical Sciences & Peking Union Medical College, Beijing, China; ^2^ Department of Medical Research Center, State Key Laboratory of Complex Severe and Rare Diseases, Peking Union Medical College Hospital, Chinese Academy of Medical Science & Peking Union Medical College, Beijing, China

**Keywords:** rescue thrombolysis, percutaneous coronary intervention, ST-elevation myocardial infarction, coronary artery ectasia, case report

## Abstract

**Background:**

Coronary artery ectasia (CAE) is a rare disease characterized by pathological ectasia of the coronary artery. In the setting of ST-segment elevation myocardial infarction (STEMI), primary percutaneous coronary intervention (PCI) of the ectatic culprit vessel is less likely to succeed due to significant thrombus burden and tortuous ectatic coronary anatomy. However, there are currently no clinical guidelines for subsequent treatment when primary PCI fails. We present a case of successful revascularization by rescue thrombolysis after failed primary PCI in a CAE patient presenting with STEMI.

**Case presentation:**

A 63-year-old male presented with a four-hour history of typical rest angina and electrocardiographic findings of inferior ST-segment elevation was diagnosed with acute inferior STEMI. Emergency coronary angiography revealed a complete mid-segment occlusion of the right coronary artery (RCA) and abnormal ectasia of the three main coronary arteries. Multiple attempts to cross the lesion with a guidewire were unsuccessful, leading to the termination of primary PCI. Subsequently, rescue thrombolysis was administered, and repeat angiography confirmed recanalization of the RCA.

**Discussion:**

This case is the first to highlight the potential benefit of timely rescue thrombolysis in CAE patients with STEMI when primary PCI fails. It provides useful clinical insight into the management of this high-risk subset of STEMI patients.

## 1 Introduction

Coronary artery ectasia (CAE) is a rare disease characterized by pathological dilation of the coronary artery, exceeding 1.5 times the diameter of adjacent normal segments (1). When percutaneous coronary intervention (PCI) is performed in CAE patients with ST-elevation myocardial infarction (STEMI), the significant thrombotic burden and distorted vascular anatomy of the ectatic coronaries may greatly impede the passage of guide wires and stent delivery (2). However, when primary PCI fails, no clinical guidelines for subsequent treatment options are available, and it is unclear whether rescue thrombolysis is effective. Here for the first time, we present a case of successful revascularization by rescue thrombolysis after failed primary PCI in an elderly male with CAE and STEMI.

## 2 Case report

**Figure 1:**
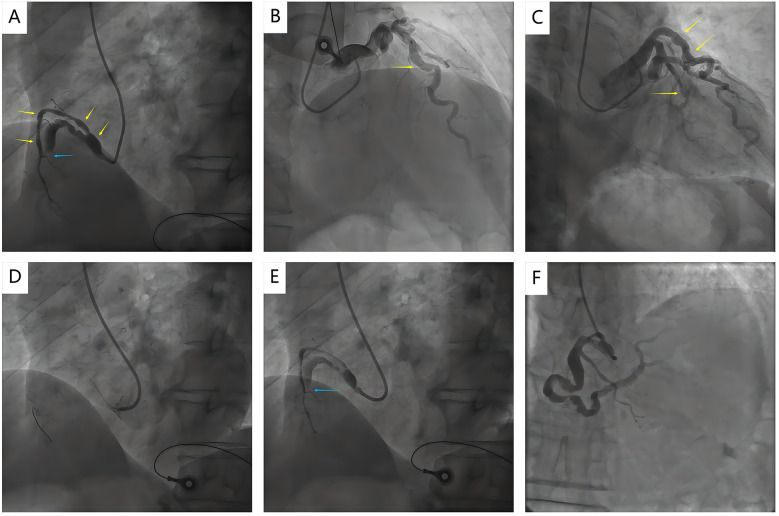
Coronary angiography images before and after rescue thrombolysis. **(A)** Coronary angiography revealed a totally occluded in the middle RCA with TIMI 0 flow (blue arrow); **(A–C)** Coronary angiography showed abnormal ectasia of the RCA, LAD, and LCX (yellow arrows); **(D, E)** Failed to cross the RCA lesion with a guidewire when Primary PCI (blue arrow); **(F)** Repeat angiography after rescue thrombolysis showed recanalization of RCA; RCA, right coronary artery; LAD, left anterior descending; LCX, left circumflex arteries; PCI, percutaneous coronary intervention.

A 63-year-old male patient presented with an acute onset of typical angina on rest. He arrived in the emergency department 4 hours later. The patient presented with a distressed facial expression. Extremities were warm and dry. Vital signs: blood pressure 152/100 mmHg, heart rate 108 bpm with regular rhythm. No crackles detected on lung auscultation. The initial electrocardiogram revealed convex upward ST-segment elevation in leads Ⅱ, Ⅲ and aVF. After comprehensive consideration of medical history and clinical examination, the cardiologist judged ‘acute inferior STEMI’ as the most likely diagnosis. The patient received aspirin, ticagrelor, and was prepared for primary PCI. Coronary angiography revealed a complete occlusion in the mid right coronary artery (RCA) (Figure 1A). RCA, left anterior descending (LAD), and left circumflex arteries (LCX) appeared ectatic (Figures 1A-C). Further history-taking revealed that the patient had a history of syphilis infection but denied any prior diagnoses of Kawasaki disease, rheumatic autoimmune diseases, connective tissue disorders, or coronary artery surgery, etc. The etiology of CAE was considered to be most likely related to syphilis infection. After adequate heparin supplementation (100U/kg), the RCA was approached by a 6F SAL 1.0 guide catheter via right radial artery access. Multiple attempts to cross the RCA lesion with a Runthrough NS guidewire failed (Figures 1D,E and Supplementary Video 1). PCI was terminated and intravenous tirofiban was initiated. However, the patient's chest pain persisted for the following hour without relief. Based on his brief symptom onset, persistent symptoms and presence of ST elevation, rescue thrombolysis was decided to pursue by the expert group after obtaining the informed consent of the patient. Considering the use of heparinization during PCI and multiple antiplatelet therapies, as well as the potential for systemic vascular involvement by syphilis (e.g., affecting intracranial or aortic aneurysms), the risk of significant bleeding is substantially elevated. Therefore, intravenous tirofiban was terminated and a reduced dose of alteplase (an initial bolus of 8 mg intravenously, followed by 42 mg administered over the subsequent 90 minutes) was given under electrocardiogram monitoring. The patient's chest pain gradually alleviated approximately 30 minutes after alteplase administration and nearly disappeared by 90 minutes, then repeat electrocardiography showed a significant regression of the elevated ST segments in leads II, III, and aVF, with a reduction of over 50%, indicating successful thrombolysis. Coronary angiography performed a week after rescue thrombolysis confirmed recanalization of the RCA (Figure 1F and Supplementary Video 2). Furthermore, coronary angiography revealed a severe distal RCA stenosis, and the proximal and distal segments of stenosis exhibited abnormal dilation and irregular caliber, making stent implantation unsuitable. Thus, drug-eluting balloon dilation was chosen. Due to the patient's financial constraints, intravascular imaging, such as intravascular ultrasound (IVUS) or optical coherence tomography (OCT), were not conducted. Following the acute phase of STEMI, the patient was maintained on long-term dual antithrombotic therapy with aspirin 100 mg once daily and rivaroxaban 20 mg once daily. During the 12-month follow-up, the patient had no recurrence of angina pectoris, and daily activities remained unrestricted. Transthoracic echocardiography re-examination revealed no new segmental ventricular wall motion abnormalities, with left ventricular ejection fraction improving from the baseline 50% to 56%.

## 3 Discussion

CAE is defined as pathological dilation of the coronary arteries caused by various etiologies, exceeding 1.5 times the diameter of adjacent normal segments (3). It is a relatively rare coronary angiographic finding, with an incidence of 0.37%–2.53% (4).

Atherosclerosis is the most common etiology of CAE, with approximately 50% of CAE cases associated with atherosclerosis, while 20%–30% of cases may be classified as congenital anomalies (5, 6). Other etiological factors include Kawasaki disease, systemic connective tissue diseases (e.g., Marfan syndrome), infectious diseases (e.g., syphilis, Epstein–Barr virus), vasculitis (e.g., polyarteritis nodosa), iatrogenic (e.g., percutaneous transluminal coronary angioplasty, stents, directional coronary atherectomy, angioplasty, and laser angioplasty) congenital anomalies, genetic factors, and idiopathic CAE (5, 6). In this case, the patient had no prior history of fever of unknown origin or Kawasaki disease, nor was there a history of coronary intervention/surgery or a family history of CAE. Serological testing revealed positive specific antibodies for syphilis, with a rapid plasma reagin (RPR) titer of 1:1. Immune-related serological markers, including autoantibodies associated with systemic vasculitis and antinuclear antibody profiles, were all negative, as was whole-genome testing. Peripheral vascular ultrasound, aortic computed tomography (CT), and cerebral magnetic resonance angiography (MRA) demonstrated abdominal aortic dilation and a basilar artery aneurysm, without significant evidence of atherosclerosis. Based on a comprehensive evaluation of the etiology of CAE, syphilis infection is considered a likely contributing factor.

Unlike other rupture-prone ectatic vascular disorders (e.g., intracranial or aortic aneurysmal dilatation), the primary clinical concern in CAE centers on thrombotic risk and subsequent myocardial infarction (7). In CAE patient presented with STEMI, PCI of an ectatic culprit artery is associated with higher rates of failure due to the presence of substantial thrombus burden and distorted vascular anatomy (3). It is widely acknowledged that immediate revascularization is critical to improve the outcome in patients with STEMI (8). Current guidelines advocate the general patients with STEMI to accept prompt PCI in experienced centers or intravenous thrombolysis. Furthermore, rescue PCI is regarded as a reasonable option after failed intravenous thrombolysis (9, 10). Nevertheless, the reverse—rescue thrombolysis, that is, implementing thrombolysis in patients with STEMI after primary PCI fails—is now not well defined. Here the CAE patient with STEMI reported in the case underwent timely intravenous thrombolysis after the initial PCI attempt failed, ultimately achieving vessel recanalization. The decision of our team to perform remedial intravenous thrombolysis after PCI failure was based on the following considerations: (1) limited expected efficacy of interventional strategy adjustment. Targeted adjustments of interventional devices (e.g., replacing catheters with stronger support or upgrading guidewires), although theoretically potentially improving the guidewire's ability to cross the lesion, would prolong the intervention time—a critical concern for STEMI patients, as delayed myocardial reperfusion increases the risk of irreversible myocardial injury and adverse events. Furthermore, the failure of PCI was primarily attributed to tortuous vascular anatomy and severe thrombus burden, which could not be effectively addressed by device upgrades alone, particularly considering that the currently used 6F SAL 1.0 guide catheter provided adequate support and the Runthrough NS guidewire exhibited good deliverability; (2) lack of mechanical thrombectomy devices. Given insufficient evidence supporting the benefit of mechanical thrombectomy (11), its use during primary PCI for STEMI patients is not currently recommended; thus, our interventional catheterization laboratory does not routinely stock mechanical thrombectomy devices; (3) Insufficient evidence for intracoronary thrombolysis. Current research on intracoronary thrombolysis is based on the premise that thrombolytics can improve coronary microcirculation in STEMI patients after emergency PCI, rather than aiming to dissolve the thrombus itself (12). Additionally, our team's prior experience with similar CAE cases has shown suboptimal efficacy of this method for thrombus clearance. In contrast, intravenous thrombolysis, as a guideline-recommended alternative to PCI, was considered the optimal choice; (4) high risk of emergency coronary artery bypass grafting (CABG). According to the multi-center study, emergency CABG is associated with 13% in-hospital mortality. Early complications included reoperation for bleeding (15%), postoperative stroke (6%) and *de novo* dialysis for acute kidney injury (6%). This patient, with underlying vascular fragility (abdominal aortic dilation, basilar artery aneurysm), was at higher potential risk for emergency CABG-related mortality and complications (e.g., bleeding, postoperative stroke) (13).

It is worth noting that the risk of thrombosis and bleeding should be fully assessed before thrombolytic therapy. In this case, the selection of half-dose alteplase thrombolysis was based on three key reasons: (1) evidence from the EARLY-MYO Trial (Early Routine Catheterization After Alteplase Fibrinolysis Versus Primary PCI in Acute ST-Segment-Elevation Myocardial Infarction), which demonstrated the efficacy and safety of half-dose alteplase in STEMI patients (14); (2) elevated bleeding risk due to multiple antiplatelet and anticoagulation therapies: the patient received a loading dose of aspirin and ticagrelor preoperatively, weight-based unfractionated heparin intraoperatively (heparin may not have been fully metabolized at the time of thrombolysis), and intravenous tirofiban after PCI failure; and (3) potential systemic vascular involvement by syphilis (15): CAE was suspected to be caused by syphilis, which may also induce multiple aneurysms, further increasing bleeding risk.

Of course, as a single case, we explicitly acknowledged the experience may not generalize. Current guidelines lack support for rescue intravenous thrombolysis in CAE-related STEMI with failed PCI. Causality and safety (e.g., syphilis-CAE link, thrombolysis risks) remain unestablished. Prospective studies are needed to determine optimal use of rescue thrombolysis and thrombolytic selection/dosage.

## 4 Conclusion

This case is the first to highlight the potential benefit of timely rescue thrombolysis in CAE patients with STEMI when primary PCI fails. It provides useful clinical insight into the management of this high-risk subset of STEMI patients.

## Data Availability

The original contributions presented in the study are included in the article/Supplementary Material, further inquiries can be directed to the corresponding authors.
